# In-depth immune profiling of a patient with immunodeficiency, centromeric instability, and facial anomalies syndrome type 2 caused by a novel mutation in *ZBTB24*

**DOI:** 10.1093/cei/uxaf016

**Published:** 2025-03-19

**Authors:** Colleen M Roark, Diana Ramírez-Vásquez, Jenniffer Yissel Giron Martinez, Xin Zhen, Alexa N Del Bene, Shannon E Gibson, Megan M Dobrose, Natasha B Halasa, Lizbeth Blancas-Galicia, Ruben Martinez-Barricarte

**Affiliations:** Division of Genetic Medicine, Department of Medicine, Vanderbilt University Medical Center, Nashville, TN, USA; Division of Molecular Pathogenesis, Department of Pathology Microbiology and Immunology, Vanderbilt University Medical Center, Vanderbilt University Medical Center, Nashville, TN, USA; Department of Immunology, Hospital de la Niñez Oaxaqueña, Oaxaca, Mexico; Department of Pediatrics, Hospital Materno Infantil San Lorenzo de los Mina, Santo Domingo, Dominican Republic; Division of Genetic Medicine, Department of Medicine, Vanderbilt University Medical Center, Nashville, TN, USA; Division of Molecular Pathogenesis, Department of Pathology Microbiology and Immunology, Vanderbilt University Medical Center, Vanderbilt University Medical Center, Nashville, TN, USA; Division of Genetic Medicine, Department of Medicine, Vanderbilt University Medical Center, Nashville, TN, USA; Division of Molecular Pathogenesis, Department of Pathology Microbiology and Immunology, Vanderbilt University Medical Center, Vanderbilt University Medical Center, Nashville, TN, USA; Division of Genetic Medicine, Department of Medicine, Vanderbilt University Medical Center, Nashville, TN, USA; Division of Molecular Pathogenesis, Department of Pathology Microbiology and Immunology, Vanderbilt University Medical Center, Vanderbilt University Medical Center, Nashville, TN, USA; Division of Genetic Medicine, Department of Medicine, Vanderbilt University Medical Center, Nashville, TN, USA; Division of Molecular Pathogenesis, Department of Pathology Microbiology and Immunology, Vanderbilt University Medical Center, Vanderbilt University Medical Center, Nashville, TN, USA; Department of Pediatrics, Vanderbilt University Medical Center, Nashville, TN, USA; Immunodeficiency Laboratory, National Institute of Pediatrics, Mexico City, Mexico; Division of Genetic Medicine, Department of Medicine, Vanderbilt University Medical Center, Nashville, TN, USA; Division of Molecular Pathogenesis, Department of Pathology Microbiology and Immunology, Vanderbilt University Medical Center, Vanderbilt University Medical Center, Nashville, TN, USA; Vanderbilt Center for Immunobiology, Vanderbilt University Medical Center, Nashville, TN, USA; Vanderbilt Genetics Institute, Vanderbilt University Medical Center, Nashville, TN, USA; Vanderbilt Institute for Infection, Immunology and Inflammation, Vanderbilt University Medical Center, Nashville, TN, USA

**Keywords:** immunodeficiency, centromeric instability, and facial anomalies (ICF) syndrome, inborn errors of immunity, mass cytometry, ZBTB24, epigenetics

## Abstract

Immunodeficiency, centromeric instability, and facial anomalies (ICF) syndrome is a rare genetic disorder characterized by recurrent, severe infections. Mutations in DNA methylation genes such as *DNMT3B* (ICF1), *ZBTB24* (ICF2), *CDCA7* (ICF3), and *HELLS* (ICF4) cause ICF. ICF2 syndrome has been previously described, yet the extent of its clinical presentation and immunological consequences needs to be further elucidated. We describe a patient with a novel homozygous mutation in *ZBTB24* (Q375Hfs*3). While infections with extracellular pathogens are frequent in other reported ICF2 patients, our patient also displays infections by intracellular pathogens. At the molecular level, we showed that the novel mutation results in a truncated ZBTB24 protein that disrupts its function in DNA methylation. We thoroughly characterized the immunological consequences of ZBTB24 deficiency using mass cytometry coupled with state-of-the-art computational methods. Our analysis revealed reduced frequencies of natural killer cells and class-switched memory B cell populations in our patient, along with low levels of the immunoglobulin isotypes IgG4 and IgM. Despite observing normal cell frequencies within the T and myeloid compartments, the clinical presentation of this patient suggests a functional defect in immune cells known to be critical to combat intracellular pathogens. Overall, this study expands the clinical and immunological features of ZBTB24 deficiency and highlights the importance of ZBTB24 to the human immune response.

## Teaser

Immunodeficiency, centromeric instability, and facial anomalies (ICF) syndrome is a rare genetic disorder characterized by recurrent, severe infections. We describe a patient with a novel homozygous mutation in ZBTB24 (Q375Hfs*3). We thoroughly characterized the immunological consequences of ZBTB24 deficiency using mass cytometry coupled with state-of-the-art computational methods. Our analysis revealed reduced frequencies of natural killer cells and class-switched memory B cell populations in our patient, along with low levels of the immunoglobulin isotypes IgG4 and IgM.

## Graphical Abstract

**Graphical Abstract:**
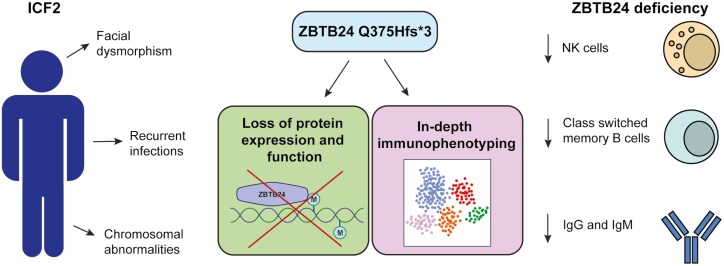

## Introduction

Inborn errors of immunity (IEIs) are a heterogeneous group of monogenic diseases. Disease-causing genetic variants alter protein expression or function in pathways essential to immune responses. Clinically, this can result in susceptibility to severe infection, autoimmunity, autoinflammatory diseases, cancer, allergy, or bone marrow failure [1, 2]. The study of IEI can lead to precise therapy for patients with these diseases. Furthermore, it can reveal previously unknown genes, proteins, and pathways critical to the development and function of the immune system [1, 3].

Immunodeficiency, centromeric instability, and facial anomalies (ICF) syndrome is an IEI typically characterized by recurrent infections, chromosomal abnormalities, and facial dysmorphism as the result of pathogenic variants in DNA methylation genes such as *DNMT3B* (ICF1) [4], *ZBTB24* (ICF2) [5], *CDCA7* (ICF3) [6], and *HELLS* (ICF4) [6]. The study of ICF has highlighted the critical and non-redundant roles of DNA methylation in human immunity. ZBTB24 deficiency, or ICF2 specifically, has been described in only a few patients. While ICF2 is rarer than ICF1, which comprises about half of all ICF cases, it is more common than ICF3 or ICF4 [7]. Typical clinical presentations of ICF2 include hypertelorism and other types of facial dysmorphism. Recurrent respiratory and gastrointestinal infections are also common in this disease due to immune dysfunction [8].

The function of ZBTB24 and its role in ICF2 when mutated are not fully understood. ZBTB24 is a member of the BTB-POZ family, containing an N-terminal BTB domain that mediates interactions with transcriptional co-regulators. It also contains zinc finger domains that can bind to DNA with sequence specificity and an AT-hook that plays a role in DNA interactions [9]. There is evidence that ZBTB24 has direct and indirect roles in immune function. It has been shown that ZBTB24 interacts with DNMT3B, the causative gene for ICF1, to control DNA methylation of ICF-related genes [10]. Additionally, ZBTB24 activates transcription of CDCA7, playing a role in regulating T cell apoptosis through the CDCA7/TRAIL-receptor pathway [11]. Loss of ZBTB24 in ICF patients also results in defective non-homologous end-joining (NHEJ), leading to impaired B cell class-switching [12]. Finally, previous work illustrates how ZBTB24 represses IRF4 and BLIMP-1 to regulate B cell proliferation [13]. These earlier findings highlight the importance of ZBTB24 to immune function, yet more remains to be understood about the relationship between ZBTB24 deficiency and its immunological consequences. In this study, we assess the functional implications of a novel *ZBTB24* mutation and perform in-depth immunophenotyping by mass cytometry in patient leukocytes to highlight the importance of ZBTB24 and DNA methylation in human immunity.

## Materials and methods

### Genetic analysis

The mutation in *ZBTB24* c.1125_1135del (p.Q375Hfs*3) was identified using the Invitae Primary Immunodeficiency Panel composed of 429 genes. Genomic DNA was isolated from the whole blood of the patient and his parents using the GeneJET Genomic DNA Purification Kit (Thermo Fisher Scientific, Waltham, MA, USA). The mutation in *ZBTB24* was confirmed by Sanger sequencing. Primers were designed to amplify the exon 4 of *ZBTB24* containing the mutation of interest (Supplementary Table S1). A PCR was set up with these primers and patient, family, and healthy donor genomic DNA using DreamTaq Green PCR Master Mix (2X) (Thermo Fisher Scientific, Waltham, MA, USA). Reactions were incubated in a thermal cycler according to product specifications and run on a 2% agarose gel using gel electrophoresis to confirm amplification. PCR products were Sanger sequenced (Genewiz Azenta Life Sciences, South Plainfield, NJ, USA) using primers in Supplementary Table S1.

### Overexpression

#### Plasmid preparation

A plasmid containing the *ZBTB24* gene (pDONR223_ZBTB24) was obtained from Addgene (Addgene, Watertown, MA, USA). We introduced the mutation Q375Hfs*3 using site-directed mutagenesis with the primers shown in Supplementary Table S1 and using PfuUltra II Fusion HS DNA Polymerase (Agilent Technologies, Santa Clara, CA, USA) following the manufacturer’s instructions. PCR products were digested with the restriction enzyme Dpnl (New England Biolabs, Ipswich, MA, USA), transformed into Stellar Competent Cells (Takara Bio, Kusatsu, Shiga, Japan), and plated on LB agar with ampicillin (100 mg/ml). The next day, colonies were incubated in LB broth supplemented with ampicillin (100 mg/ml). A miniprep was performed using the GeneJET Plasmid Miniprep Kit (Thermo Fisher Scientific, Waltham, MA, USA). The plasmid sequence was confirmed with Sanger sequencing (Genewiz Azenta Life Sciences, South Plainfield, NJ, USA) using primers in Supplementary Table S1. We amplified the coding sequence of *ZBTB24* cDNA with primers that introduce a C-terminal histidine tag shown in Supplementary Table S1 in the wild type and mutant by PCR using the Platinum II Hot-Start Green PCR Master Mix (2X) (Thermo Fisher Scientific, Waltham, MA, USA) following manufacturer’s instructions. PCR products were then digested with Dpnl (New England Biolabs) and run on a 1% agarose gel. Bands were extracted and purified using the GeneJET Gel Extraction Kit (Thermo Fisher Scientific, Waltham, MA, USA). The gel-extracted DNA products, containing the wildtype and mutant ZBTB24 plasmids with C-terminal His-tags, were cloned into pcDNA3.1 using the pcDNA™3.1-TOPO™ TA Cloning™ Kit (Invitrogen, Waltham, MA, USA). Cloning reactions were transformed into DH5α competent cells (Thermo Fisher Scientific, Waltham, MA, USA) and plated on LB agar with ampicillin (100 mg/ml). The next day, colonies were incubated in LB broth with ampicillin (100 mg/ml). Plasmid DNA was isolated using the GeneJET Plasmid Mini Prep Kit (Thermo Fisher Scientific, Waltham, MA, USA). Plasmid sequences were confirmed with Sanger sequencing (Genewiz Azenta Life Sciences, South Plainfield, NJ, USA) using primers in Supplementary Table S1.

#### Transfection and western blot

Human Embryonic Kidney 293 (HEK293) cells were grown in Dulbecco’s Modified Eagle Medium (DMEM) with 10% fetal bovine serum (FBS). A total of 500,000 cells were seeded in six-well plates the day before transfection. Cells were either left untransfected, or they were transfected with an empty plasmid or plasmids containing the wildtype and mutant *ZBTB24* gene with C-terminal His tags using Lipofectamine 2000 Reagent (Thermo Fisher Scientific, Waltham, MA, USA) according to product specifications. Forty-eight hours after transfection, cells were collected and lysed with RIPA buffer containing protease inhibitors (Pierce Protease Inhibitor Tablets, Thermo Fisher Scientific, Waltham, MA, USA). Cell lysates were quantified using the Detergent Compatible Protein Assay (Bio-Rad, Hercules, CA, USA). A total of 5 μg of protein from each condition were separated via sodium dodecyl sulfate-polyacrylamide gel electrophoresis (SDS-PAGE) and transferred onto a polyvinylidene fluoride (PVDF) membrane (MilliporeSigma, Burlington, MA, USA). The membrane was probed with an anti-ZBTB24 rabbit polyclonal antibody (Proteintech, Rosemont, IL, USA), followed by a secondary anti-rabbit goat IgG HRP conjugated antibody (AP156P, MilliporeSigma, Burlington, MA, USA). The membrane was stripped and re-probed with an anti-His mouse antibody (J099B12, Biolegend, San Diego, CA, USA), as well as an anti-GAPDH mouse antibody (HRP-60004, Proteintech, Rosemont, IL, USA) as a loading control. Then, a secondary anti-mouse goat IgG HRP conjugated antibody (AP127P, MilliporeSigma, Burlington, MA, USA) was used. Membranes were developed with Pierce ECL Western Blotting Substrate (Thermo Fisher Scientific, Waltham, MA, USA).

#### RT-qPCR

Transfected cells were collected and pelleted. RNA was extracted using the Qiagen RNeasy Plus Mini Kit (Qiagen, Hilden, Germany). RT-qPCR was performed using the Luna Universal One-Step RT-qPCR Kit (New England Biolabs, Ipswich, MA, USA) on the Bio-Rad CFX Real-Time PCR machine, which uses CFX Maestro Software (Bio-Rad, Hercules, CA, USA) to calculate Cq values. *ZBTB24* expression was calculated relative to *GUS* expression. Primers used to amplify *ZBTB24* and *GUS* are found in Supplementary Table S1.

### Bisulfite sequencing

Bisulfite conversion of DNA was performed using the EZ DNA Methylation Kit (Zymo Research, Irvine, CA, USA). A PCR was set up with *SNORD115-14* primers (Supplementary Table S1), and the bisulfite converted DNA using Platinum II Hot-Start Green PCR Master Mix (2X) (Thermo Fisher Scientific, Waltham, MA, USA) following the manufacturer’s instructions. PCR products were then cloned into pcDNA3.1 using the pcDNA3.1/V5-His TOPO TA Expression Kit (Invitrogen, Waltham, MA, USA). Cloning reactions were transformed into DH5α competent cells (Thermo Fisher Scientific, Waltham, MA, USA) or Stellar competent cells (Takara Bio, Kusatsu, Shiga, Japan) and plated on LB agar with ampicillin. Colonies were incubated in LB broth and ampicillin (100 mg/ml). Plasmid DNA was isolated using the GeneJET Plasmid Mini Prep Kit (Thermo Fisher Scientific, Waltham, MA, USA). Plasmids were Sanger sequenced (Genewiz Azenta Life Sciences, South Plainfield, NJ, USA) using primers in Supplementary Table S1 and compared to the unconverted sequence to determine the percent methylation of the sequence. Frequencies of CpG methylation were analyzed using Prism 10 (GraphPad Software, San Diego, CA). *P*-values were calculated using a one-way ANOVA followed by Tukey’s multiple comparisons test.

### Mass cytometry

Human peripheral blood mononuclear cells (PBMCs) were isolated by Ficoll–Hypaque density gradient centrifugation (Amersham-Pharmacia-Biotech, Buckinghamshire, UK) from whole-blood samples obtained from the patient, parents, and healthy donors and cryopreserved in FBS containing 10% DMSO (VWR, Radnor, PA, USA). Staining, data acquisition, and analysis were performed as described previously [14].

### Antibody isotyping

Antibody isotyping was performed using the ProcartaPlex™ Human Antibody Isotyping Panel, 7plex kit (Invitrogen, Waltham, MA, USA) on patient, parent, and healthy control serum according to product specifications.

## Results

### Novel *ZBTB24* variant in a patient with ICF2

**Figure 1:**
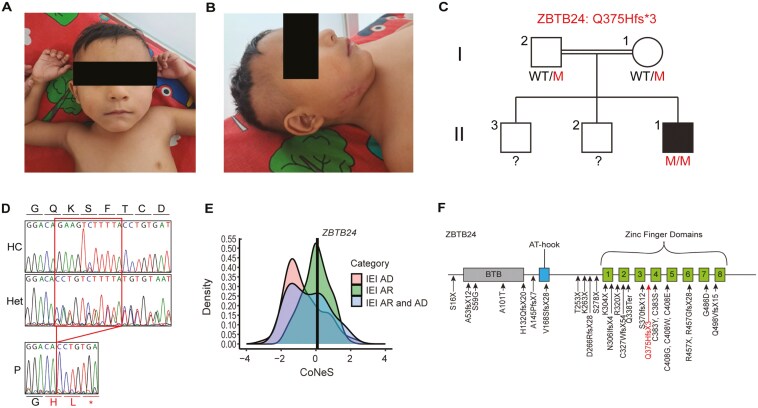
Novel ZBTB24 deficiency. (A) The frontal view of the patient shows a broad forehead, hypertelorism, and inverse epicanthus. (B) The lateral view shows a bulbous nose and scars at the site of the ulcerated adenitis. (C) Familial segregation of the ZBTB24 mutation Q375Hfs*3. (D) Sanger sequencing results of healthy donors (HC), a parent (Het), and the patient (P) of the region of exon containing the ZBTB24 mutation. The amino acid consensus for wildtype ZBTB24 is above the sequences, while the frameshift and predicted premature stop codon introduced by the ZBTB24 mutation is below the sequences. (E) Distribution of consensus negative selection (CoNeS) scores for autosomal dominant (AD) and autosomal recessive (AR) genes underlying inborn errors of immunity (IEI). The vertical line represents *ZBTB24*. (F) Protein map of ZBTB24 showing its known mutations and the mutation Q375Hfs*3.

We describe a 4-year and 9-month-old male patient presenting with ICF syndrome coming from consanguineous parents from an endogamous community in southwestern Mexico. The patient received the BCG vaccine without adverse effects but presented with delayed language and psychomotor development from birth and a broad forehead, hypertelorism, bulbous nose, inverse epicanthus, strabismus, and clubfoot (Fig. 1A). The patient required several hospitalizations for diarrhea and fever, where *Giardia lamblia* infection was discovered at 14 months of age. At 2 years and 8 months of age, he presented with ulcerative submaxillary adenitis (Fig. 1B), which resulted in hospitalization after his condition failed to improve with oral antibiotics. The wound was positive for *Staphylococcus aureus*, and the patient received clindamycin and cefotaxime intravenously. The wound also presented with dehiscence, leading to a lymph node biopsy that revealed multiple granulomas associated with multinucleated Langhans giant cells with central caseous necrosis. These findings were consistent with possible *Mycobacterium tuberculosis* [15], and since the patient lived in an *M. tuberculosis* endemic region, antituberculosis treatment with isoniazid, rifampicin, ethambutol, and pyrazinamide was given for nine months with a favorable response. These clinical findings provided evidence of an *M. tuberculosis* episode according to the Graham criteria, which assess histological features and the patient’s response to treatment [15]. The clinical evolution indicated a potential inborn error of immunity and was consistent with ICF syndrome. Paraclinical studies were thus requested, finding IgG and IgM below normal ranges. By Invitae Primary Immunodeficiency Gene Panel, we identified a novel homozygous 11 nucleotide deletion in exon 4 of the gene encoding zinc finger and BTB domain containing 24 (*ZBTB24*) (c.1125_1135del). The mutation segregates as an autosomal recessive trait, and the family history shows two healthy older brothers, ages 14 and 17 (Fig. 1C). The mutation introduces a frameshift that leads to a predicted premature stop codon three amino acids after the mutation (p.Q375Hfs*3, Fig. 1D). The consensus-based measure of negative selection (CoNeS) score of *ZBTB24* falls at the peak of genes known to cause autosomal recessive inborn errors of immunity [16] (Fig. 1E). Previously reported *ZBTB24* mutations span the length of the gene, resulting in ICF2. The mutation we identified falls in the zinc finger domains of the translated protein (Fig. 1D and F). These results suggest that this novel mutation may lead to disruption of ZBTB24 protein expression or function, contributing to the clinical features of the patient.

### Q375Hfs*3 impairs ZBTB24 protein expression and alters DNA methylation

**Figure 2:**
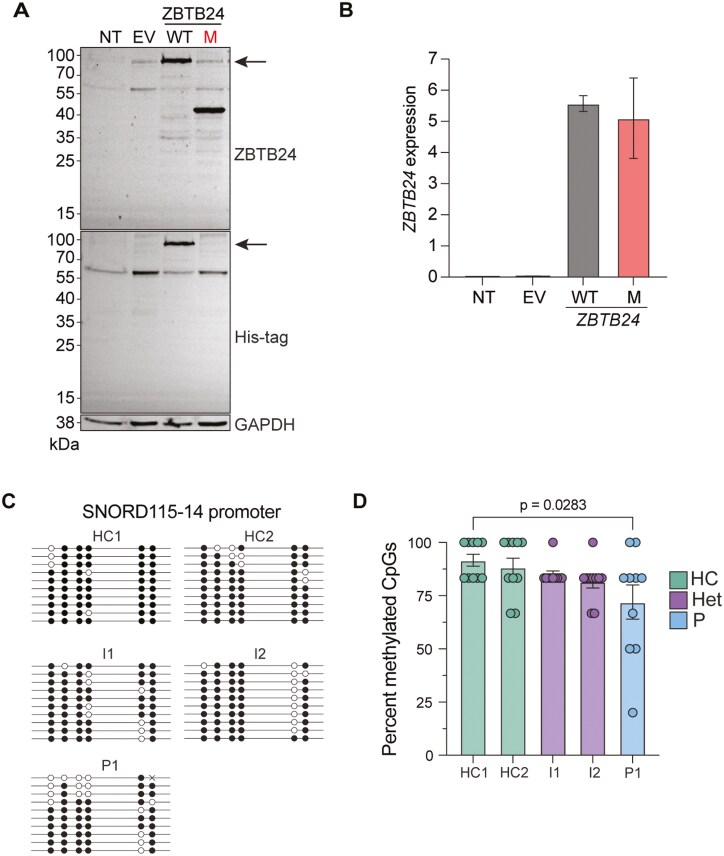
Q375Hfs*3 alters ZBTB24 expression and function. HEK293 cells were either left non-transfected (NT) or were transfected with an empty vector (EV), or plasmids containing WT or mutant (M) ZBTB24 C-terminally His tagged. (A) Western blot of protein isolated from HEK293 cells showing ZBTB24 expression with a molecular weight of 78 kDa, indicated by black arrows. GAPDH was used as a loading control. An experiment representative of 3. (B) qPCR of mRNA isolated from HEK293 cells. ZBTB24 expression was calculated relative to GUS expression. An experiment representative of 3. (C) Bisulfite sequencing of genomic DNA from patients, parents, and healthy donors. Black dots represent methylated CpG sites, and white dots represent unmethylated CpG sites. Each line represents the sequence of a single clone (*n* = 10). (D) Quantification of methylated CpG sites. Error bars represent the standard error of the mean. *P*-values were calculated using a one-way ANOVA followed by Tukey’s multiple comparisons test.

Given that the mutation Q375Hfs*3 leads to a predicted premature stop codon, we tested its effect on protein expression. We overexpressed a C terminally His-tagged version of wildtype and mutant *ZBTB24* in HEK293 cells. We performed western blotting using an antibody specific for the AT-hook and the first two zinc finger domains of ZBTB24, N-terminal of the mutation. ZBTB24 has a molecular weight of 78 kDa and we observed a lower molecular weight band of about 42.5 kDa in the mutant, consistent with the predicted molecular weight of the mutant ZBTB24 protein (Fig. 1F). Using an anti-His tag antibody, we could not detect any band on the cells transfected with the mutant allele, suggesting no reinitiation after the premature stop codon caused by the mutation (Fig. 2A). We assessed *ZBTB24* mRNA expression by reverse transcriptase quantitative PCR (RT-qPCR), which confirmed the overexpression of *ZBTB24* in HEK293 cells and indicated that, at least in this system, there is no nonsense-mediated mRNA decay (Fig. 2B). We next wanted to study if this mutation led to a loss of function even if the truncated protein was expressed. ZBTB24 regulates DNA methylation in conjunction with DNA methyl transferase 3B (DNMT3B) [10]. To test the effect of this mutation on the function of ZBTB24 in DNA methylation, we studied methylation of the promoter region of the small nucleolar RNA gene *SNORD115-14* previously described as a DNA methylation target of ZBTB24 [17] (Fig. 2C and D). Using patient, heterozygote, and healthy control DNA, we performed bisulfite sequencing on this target and quantified methylation as a percentage of the six total CpG sites in a region of the *SNORD115-14* promoter to assess the impact of the *ZBTB24* mutation on DNA methylation. While no significant difference was observed in CpG methylation between healthy donors and the heterozygous parents, there was a decrease in methylation in patient DNA as compared to healthy donors, suggesting the *ZBTB24* Q375Hfs*3 mutation leads to a loss of function. These data show that the Q375Hfs*3 mutation disrupts ZBTB24 protein expression and function in DNA methylation, aligning with previously reported ZBTB24 deficiencies that lead to ICF2.

### Decreased natural killer cell frequencies in ZBTB24 deficiency caused by Q375Hfs*3

**Figure 3:**
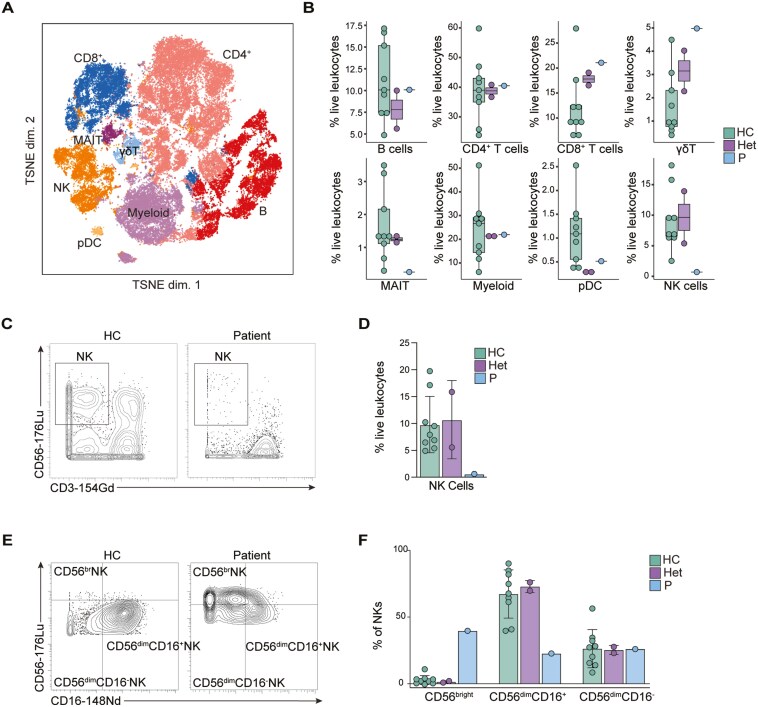
Leukocyte immunophenotyping. (A) Dimensional reduction by t-SNE of the 33 cell surface markers used for mass cytometry immunophenotyping. Each color represents a distinct immune cell type identified by manual clustering. A total of 10,000 cells from healthy controls, heterozygote carriers, and the patient are shown. (B) Frequencies of immune cell populations as a percentage of total leukocytes for healthy controls (HC), heterozygous carriers (Het), and the patient (P). (C) Representative gating of CD56 vs CD3 for natural killer (NK) cells from one healthy control (HC) and the patient (P). Gating strategy can be found in Supplementary Fig. S1. (D) Frequencies of NK cells as a percentage of live leukocytes in healthy controls (HC), heterozygous carriers (Het), and the patient (P) obtained from manual gating. (E) Representative gating of CD56 vs CD16 for natural killer (NK) cells from one healthy control (HC) and the patient (P). Gating strategy can be found in Supplementary Fig. S1. (F) Frequencies of NK cell subpopulations as a percentage of NK cells in healthy controls (HC), heterozygous carriers (Het), and the patient (P) obtained from manual gating.

Immunodeficiency is characteristic of ICF syndrome, yet specific phenotypes are variable across different ICF subtypes, mutation locations, and patients (Table 1). To understand the consequences of the mutation Q375Hfs*3 in the overall architecture of the immune system, we performed in-depth immunophenotyping by mass cytometry on PBMCs of the patient, parents, and healthy donors. We used our previously published antibody panel containing 33 antibodies against common surface markers of leukocyte populations [14]. Using unsupervised clustering and dimensional reduction via t-SNE, we could identify the main immune cell populations (B cells, CD4^+^ and CD8^+^ T cells, γδ T cells, mucosal-associated invariant T cells (MAIT), myeloid cells, plasmacytoid dendritic cells (pDCs), and natural killer (NK) cells) (Fig. 3A). We observed that B cells, CD4^+^, and CD8^+^ T cells, γδ T cells, MAIT, myeloid cells, and pDC were present in the patient at frequencies similar to healthy controls. However, we showed a dramatic reduction in the frequency of total NK cells consistent with previous findings [22, 24, 27, 28, 33, 35, 38, 39]. This phenotype was confirmed using manual gating (Fig. 3C and D). To determine the effect of the mutation on NK cell development, we examined NK subsets using CD56 and CD16 expression by manual gating. We found that the patient showed an accumulation of CD56^bright^ NK cells and a reduction in CD56^dim^CD16^+^ NK cells compared to healthy controls (Fig. 3E and F). Along with a decrease in total NK cells, this indicates that ZBTB24 deficiency results in developmental arrest in an immature state of NK cells (CD56^bright^) and fewer mature, highly cytotoxic NK cells (CD56^dim^CD16^+^) [40]. Together, this provides further evidence that ZBTB24 is critical for NK differentiation or survival.

**Table 1:** Clinical phenotypes of ZBTB24 deficiency. Summary of the genetic, clinical, and immunological features of reported *ZBTB24* mutations.

Case	Sex	Immunological findings	Infections	Age at last reporting	Dead or alive	Origin	Mutation	Inheritance	Other clinical features	Reference
1	F	Panhypogammaglobulinemia	Bronchopneumonia, Pneumocystis jirovecii pneumonia, Candida	13	Dead	Scotland	c.47C > G p.S16*	Homozygous	Flattened nasal bridge, triangular face, upturned nose, frontal bossing sparse hair	[8, 18]
2	M	Agammaglobulinemia, reduced T cell proliferation to PAH/sIL2	Bronchopneumonia, Pneumocystis jirovecii	13		Turkey	c.501dup p.V168Sfs*28	Homozygous		[8, 18]
3	M	Panhypogammaglobulinemia				Turkey	c.759C > G p.T253*	Homozygous	Hypertelorism, hypospadias, brain with distinct bilateral small areas suspicious of focal cortical heterotopy	[5, 8]
4	M	Panhypogammaglobulinemia	Several bacterial infections, meningoencephalitis	4.5	Dead	Turkey	c.759C > G p.T253* (suspected)	Homozygous	Hypertelorism, hypospadias, umbilical hernia	[5]
5	M	Severe hypogammaglobulinemia	Pyelonephritis, recurrent otitis, pneumonia, gastroenteritis, recurrent impetigo	7	Dead	Germany	c.833C > G/ 1222T > G p.S278*/ C408G	Compound heterozygous	Broad nasal bridge, hypertelorism, flat philtrum, Hodgkin lymphoma	[8, 18, 19]
6	F	Hypogammaglobulinemia	Bronchopneumonia			Germany	c.833C > G/ 1222T > G p.S278*/ C408G	Compound heterozygous	Broad nasal bridge, hypertelorism, flat philtrum	[8, 18, 19]
7	M	Agammaglobulinemia, reduced T cell proliferation to PAH/sIL2	Respiratory infections, candida			Turkey	c.917delA p.N306Ifs*4	Homozygous		[8, 18]
8	F	Panhypogammaglobulinemia	Bronchopneumonia, pseudomonas sepsis		Dead		c.958C > T p.R320*	Homozygous		[8, 18]
9	M	Low IgG and IgA, low CD4^+^ T cells, low B cells	Bronchopneumonia, recurrent bronchiolitis			Italy	c.1369C > T p.R457*	Homozygous	Failure to thrive; epicanthus, hypertelorism, flat nasal bridge, low set ears	[8, 18]
10	M	Panhypogammaglobulinemia, reduced T cell proliferation to PAH/sIL2				Turkey	c.958C > T p.R320*	Homozygous		[8]
11	M	No memory B cells, low IgG and IgM, elevated IgG3, high naive/memory CD4^+^ T cell ratio		13		Lebanon	c.396_397delTA p.H132Qfs*19	Homozygous	Round face, everted lower lips, high-arched palate, small chin retrognathia	[8, 20]
12	M	No memory B cells, low IgG and IgM, elevated IgG3, high naive/memory CD4^+^ T cell ratio		12		Lebanon	c.396_397delTA p.H132Qfs*19	Homozygous	Round face, everted lower lips, high-arched palate, small chin retrognathia	[8, 20]
13	M	No memory B cells, low IgG and IgM, elevated IgG3, high naive/memory CD4^+^ T cell ratio		7		Lebanon	c.396_397delTA p.H132Qfs*19	Homozygous	Round face, everted lower lips, high-arched palate, small chin retrognathia	[8, 20]
14	M	IgM levels reduced	None	8		Morocco	c.1222T > G p.C408G	Homozygous	Mild dysmorphic features	[8, 21]
15	F	Profound panhypogammaglobulinemia, reduced memory B cells in peripheral blood, and a variable cellular deficiency	ENT infections (*S. pneumoniae*), Candidiasis	18		Cape Verde	c.787A > T/ 980_981delGT p.C327Wfs*54/ K263*	Compound heterozygous	GI signs	[22, 23]
16	F	Low IgG and IgM, low B and NK cell counts reduced PAH, anti-CD3, and antigen-induced lymphocyte proliferation, low NK cell cytotoxicity	Recurrent upper airway infections and pneumonia (*E. cloacae*) recurrent protracted diarrhea (enteropathogenic *E. coli*), prolonged skin infection (*S. pyogenes*)	9		Germany	c.1222T > G p.C408G	Homozygous	Multiple facial anomalies, clubbing of fingers and toes, fused teeth, intellectual disability	[24]
17	M	Low IgM and low IgG	Recurrent respiratory tract infections, protracted CMV infection, EBV-induced hemophagocytic lymphohistiocytosis	11		Turkey	c.958C > T p.R320*	Homozygous	Café-au-lait spots, sparse hair, hemophagocytic lymphohistiocytosis, hypertelorism, broad nasal bridge, long philtrum, small low-set ears	[25]
18						India	c.1457G > A p.G486N	Homozygous	Congenital anomalies of the kidneys and urinary tract, intellectual disability, growth retardation, and deafness	[26]
19	M	Panhypogammaglobulinemia, low B cell counts	Bronchopneumonia, Candida, CMV-Infection, *Pneumocystis jirovecii* pneumonia	16		Turkey	c.917delA p.N306Ifs*4	Homozygous	Hypertelorism, flat nasal bridge, epicanthus, up-turned nose, macroglossia, telecanthus, micrognathia, low-set ears, round face	[7]
20	M	Panhypogammaglobulinemia, low B cell counts	Bronchopneumonia	5		Turkey	c.917delA p.N306Ifs*4	Homozygous	Hypertelorism, flat nasal bridge, epicanthus, up-turned nose, telecanthus, micrognathia, low-set ears, round face	[7]
21	M	Panhypogammaglobulinemia, low B cell counts	Bronchopneumonia, meningitis (*S. pneumoniae*)	16		Turkey	c.917delA p.N306Ifs*4	Homozygous	Hypertelorism, flat nasal bridge, up-turned nose, macroglossia, telecanthus, round face	[7]
22	M	Low IgM and IgG	Otitis, bronchopneumonia	32			c.909dup p.K304*	Homozygous	Hypertelorism, flat nasal bridge, epicanthus, telecanthus, micrognathia, seizures	[7]
23	M	Panhypogammaglobulinemia, low B cell counts	Bronchopneumonia, meningitis (*S. pneumoniae*)	22			Homozygous deletion on chr. 6, including *ZBTB24*	Homozygous	Hypertelorism, flat nasal bridge, epicanthus, micrognathia, low-set ears, seizures	[7]
24	M	IgG2, IgA, and IgM decreased, IgG3 increased, memory B cells very low, NK activity low	Recurrent respiratory tract infections	7	Dead	Japan	c.1148G > A p.C383Y	Homozygous	Macrocephaly, hypertelorism, epicanthal folds, midface flatness, low nasal root, long and flat philtrum, thick lips, hypoplastic primary teeth and bilateral hydronephrosis, skin abnormalities, refractory diarrhea	[22, 27]
25	M	IgG, IgG1, IgG2, IgA, and IgM decreased, B cells decreased, natural killer cell activity and lymphocyte blastoid transformation count decreased	Recurrent pneumonia and sinusitis	41	Dead	Japan	c.958C > T p.R320*	Homozygous	Ingravescence of tremor, articulation disorder and left hemiplegia, ambulation difficulty, increased tendon reflex, Babinski’s reflex and vesicorectal disorders, cortical atrophy, progressive multifocal leukoencephalopathy	[22, 27]
26	F	Low IgG and IgA, low CD27^+^ memory B cells	Recurrent pneumonia	12		Japan	c.1396C > T p.R457*	Homozygous	Hypertelorism	[27]
27	M	Panhypogammaglobulinemia, low IgM^+^ CD27^+^ memory B cells, low NK cell count	Recurrent pneumonia, sinusitis	28		Japan	c.1108_1109dup p.S370Kfs*12	Homozygous	Hypertelorism, broad flat nasal bridge	[27]
28	M	Total hypogammaglobulinemia, decreased production and maturation of B cells, decreased natural killer cells	Serious infections, including multiple episodes of febrile pneumonia, acute otitis media	17 months			c.1492-1493delCA p.Q498Vfs*15	Homozygous	Hypertelorism, epicanthal folds, low set posteriorly rotated ears, developmental delay, perianal fistula, chronic colitis	[28]
29	F	Low IgM, low B cells	Bronchopneumonia			Saudi Arabia	c.1492_1493del	Homozygous	Hypertelorism, flat nasal bridge, telecanthus.	[29]
30	F	Low IgM and IgA, low CD8^+^ T cells, low B cells	Bronchopneumonia			Saudi Arabia	c.1492_1493del	Homozygous	Hypertelorism, flat nasal bridge, telecanthus.	[29]
31	M	Low IgM and IgA, low B cells	Bronchopneumonia			Saudi Arabia	c.1492_1493del	Homozygous	Hypertelorism, flat nasal bridge, diarrhea	[29]
32	F	Hypogammaglobulinemia	Respiratory tract infection	33		Iran	p.C408E	Homozygous		[30, 31]
33	F	Hypogammaglobulinemia	Respiratory tract infection	7		Iran	p.N266Rfs*28	Homozygous		[30, 31]
34	M	Hypogammaglobulinemia	Respiratory tract infection	17		Iran	p.C383S	Homozygous		[30, 31]
35	M	Hypogammaglobulinemia	Respiratory tract infection	41		Iran	p.C383S	Homozygous		[30, 31]
36	F	Hypogammaglobulinemia	Respiratory tract infection	39		Iran	p.C383S	Homozygous		[30, 31]
37	M	Low IgG and IgM, low CD19^+^ cells, increased CD8^+^ T cell count	Recurrent stomatitis and bronchopneumonias	27		Italy	c.909dup/ 175A > G p.K304*/ S59G	Compound heterozygous	Dolichocephaly, high forehead, hypertelorism/telecanthus, cryptorchidism, chronic autoimmune cholangitis, EBV-related Hodgkin-lymphoma	[32]
38	M	Low IgG and IgM, low CD19^+^ cells, low NK cells, and function	Sepsis after birth, three pneumonias (*H. influenzae*), recurrent upper respiratory tract infections (bronchitis, otitis media, sinusitis)	28		Germany	c.1222T > G p.C408G	Homozygous	Atopic dermatitis	[33]
39	F	Low IgM and IgG4, low CD19^+^CD27^+^ cells, low NK cells, and function	Atypical mycobacteriosis, recurrent shingles, prolonged fever after vaccination with MMR	24		Germany	c.1222T > G p.C408G	Homozygous	Atopic dermatitis, scoliosis, nail clubbing, focal bronchial malformation with absence of bronchial glands and cartilage	[33]
40	F	Low IgM and IgG4, low CD19^+^CD27^+^ cells, low NK cells, and function	None	24		Germany	c.1222T > G p.C408G	Homozygous	Atopic dermatitis, idiopathic epileptic seizures	[33]
41	F	Hypogammaglobulinemia with low B cell numbers, decreased switched and unswitched memory B cells and reversal of CD4/CD8 T-cell ratio	Recurrent febrile illnesses	7		Bangladesh	c.433_434delGC p.A145Pfs*7	Homozygous	Hepatomegaly, splenomegaly, and facial dysmorphism	[34]
42	M	Severe T and NK-cell lymphopenia, B-cell lymphopenia, low post-germinal class-switched memory B cells	High EBV load	5	Dead		c.301G > A p.A101T	Homozygous	Epilepsy, severe psychomotor retardation, microcephaly, micrognathia, hypertelorism, low-set ears, epicanthal folds, and macroglossia	[35]
43			Upper respiratory tract infections	5			c.795dupA p.N266Rfs*28	Homozygous	Enteropathy, seizures	[36]
44			Upper respiratory tract infections	22			c.1148G > C p.C383S	Homozygous	Nystagmus	[36]
45			Upper respiratory tract infections, lower respiratory tract infections	8			c.1148G > C p.C383S	Homozygous	Hepatosplenomegaly	[36]
46			Upper respiratory tract infections	1.5			c.1224C > G p.C408W	Homozygous	Enteropathy	[36]
47	F	Decreased IgG, IgA, and IgM	Lower respiratory tract infections, pneumonia, and sinusitis	34		Iran	c.795_796insA	Homozygous	Paroxysmal nocturnal dyspnea (PND) and sputum cough	[37]
48	M	Decreased IgG, IgA, IgM, decreased NK cell count, increased naïve B cells, decreased CD4^+^ T-cell proliferation	Recurrent otitis media, recurrent bronchiolitis, recurrent pneumonia, CMV viremia	20			c.156delA p.A53Pfs*12	Homozygous	Round face, frontal bossing, hypertelorism, broad flat nasal bridge, prominent front teeth, strabismus, malar hypoplasia, double-row teeth	[38]
49	F	Decreased IgG, IgM, decreased NK cell count, reduced B cell count, inverted CD4^+^/CD8^+^ T-cell ratio, decreased thymic emigrant T cells, reduced memory B cells	Recurrent bronchiolitis, recurrent pneumonia	19			c.1012C > T p.Q338*	Homozygous	Hypertelorism, broad flat nasal bridge, high forehead, strabismus, telecanthus	[38]
50	F	Low CD4^+^ and CD8^+^ T cells, low B cells, low NK cells, decreased IgG, IgA, and IgM	Pneumonia	6		Turkey	c.1121–2A > T	Homozygous	Facial dysmorphism, cholelithiasis	[39]
51	M	Low CD4^+^ and CD8^+^ T cells, low B cells, low NK cells, decreased IgG, IgA, and IgM	Pneumonia	4		Turkey	c.1121–2A > T	Homozygous	Facial dysmorphism, cholelithiasis	[39]
52	M	Elevated IgG1 and IgG3, low IgG4 and IgM, low switched memory B cells, low NK cell count	Possible *M. tuberculosis*, *Giardia lamblia*, *S. aureus*	4	alive	Mexico	c.1125_1135del p.Q375Hfs*3	Homozygous	Broad forehead, hypertelorism, strabismus, inverse epicanthus, bulbous nose, ulcerated adenitis	This work

### ZBTB24 deficiency leads to reduced frequencies of class-switched memory B cells

**Figure 4:**
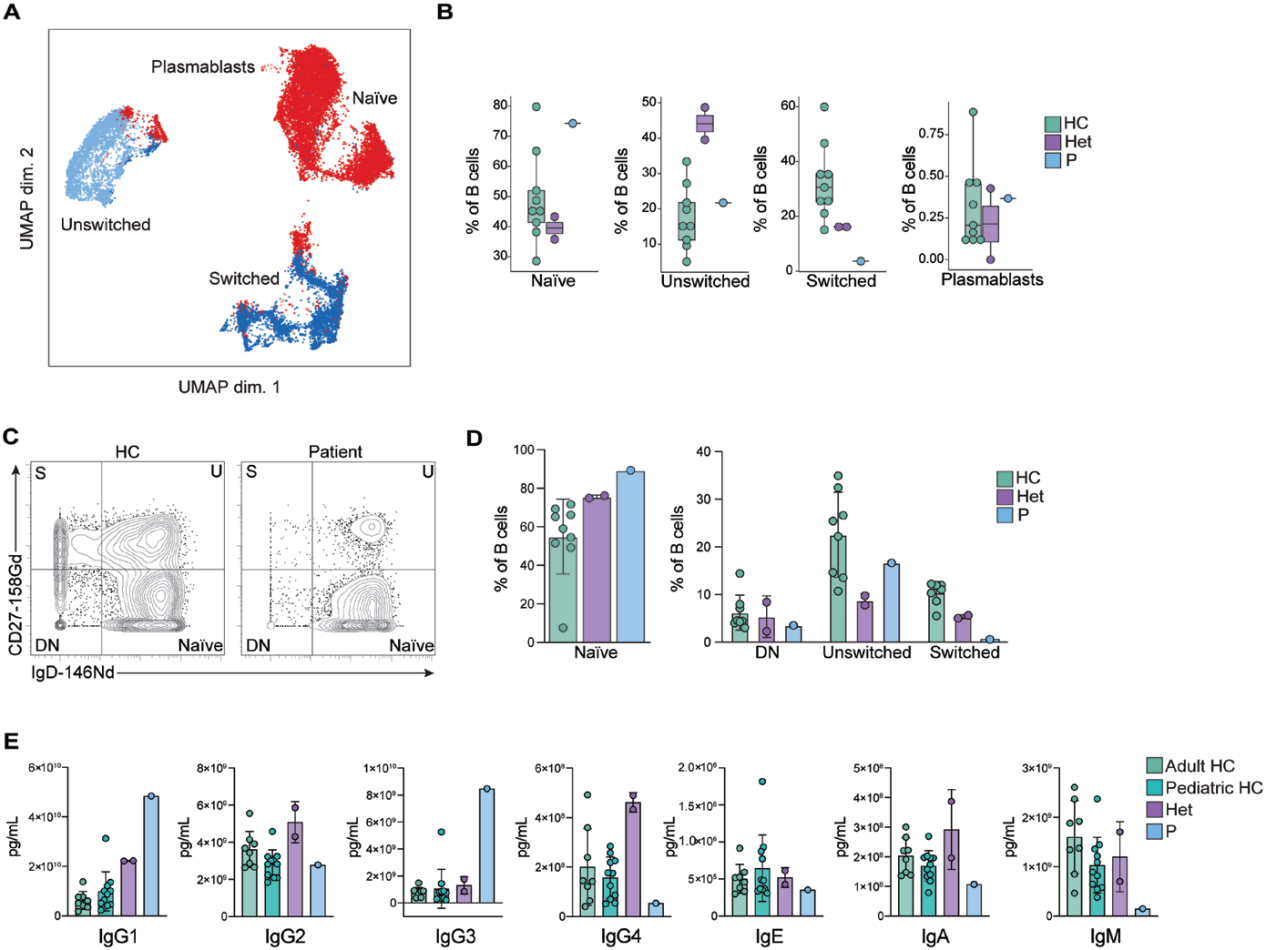
B cell immunophenotyping. (A) UMAP visualization of B cell populations. Each color represents a distinct B cell subtype identified by manual clustering. A total of 10,000 cells from healthy controls, heterozygote carriers, and the patient are shown. (B) Frequencies of B cell subpopulations as a percentage of total B cells for healthy controls (HC), heterozygous carriers (Het), and the patient (P). (C) Representative gating of CD27 vs IgD for B cells from one healthy control (HC) and the patient (P). Gating strategy can be found in Supplementary Fig. S1. (D) Frequencies of B cell subpopulations as a percentage of B cells in healthy controls (HC), heterozygous carriers (Het), and the patient (P) obtained from manual gating in FlowJo. (E) Antibody isotyping of serum from adult and pediatric healthy controls, heterozygote carriers, and the patient.

While the frequencies of the rest of the main immune populations were unchanged in the patient (Fig. 3), we studied if the distribution within each population was altered. We reclustered the B cell subpopulation and performed unsupervised clustering to further analyze the B cell compartment (Fig. 4A). Unsupervised clustering followed by dimensional reduction by UMAP revealed four distinct clusters within the B cell population, which were categorized as naïve B cells, unswitched B cells, switched B cells, and plasmablasts through manual clustering (Fig. 4A). Analysing the frequencies of these populations highlighted that the levels of patient naïve and unswitched B cells and plasmablasts were similar to those of healthy donors (Fig. 4B). However, the frequency of patient class-switched B cells was dramatically reduced compared to healthy controls and heterozygote samples. We corroborated this defect by manual gating (Fig. 4C and D). Previously described cases of ICF2 and ZBTB24 deficiency report defective class switching of B cells, resulting in decreased levels of class-switched memory B cells [8, 20, 22, 23, 27, 33–35, 38]. Therefore, our results may be consistent with a defect in class switching. To determine whether reduced class-switched B cells resulted in decreased levels of the different antibody isotypes, we performed antibody isotyping on the serum of adult and pediatric healthy controls, the parents, and the patient (Fig. 4E). Antibody isotyping revealed that the patient had similar IgG2, IgE, and IgA levels compared to the healthy controls. The levels of IgG1 and IgG3 were elevated compared to healthy controls, while IgG4 and IgM were reduced. These results are consistent with other reported ICF2 patients, who showed elevated levels of IgG3 [8, 20, 22, 27]. Reduced levels of IgM are also typical among ICF2 patients [5, 7, 8, 18, 20–25, 27–29, 32, 33, 37–39] (Table 1). While class switching is not completely impaired in the patient, it has been shown that ICF is a progressive disease, possibly leading to the depletion of B cells, and consequently all antibody isotypes, over time [24, 33, 41]. This suggests that while the patient does not present with complete hypogammaglobulinemia currently, the immune phenotype could become more severe with age. These results, therefore, enhance the evidence suggesting that reduced class-switched B cells can account for the hypogammaglobulinemia seen in ZBTB24 deficient ICF2 patients.

### Immunophenotyping does not entirely explain the clinical presentation of the patient

Due to the heterogeneous immune phenotypes seen in reported ICF2 patients and to characterize further the effect of the mutation Q375Hfs*3 on the structure of different immune populations, we performed manual gating on our CyTOF data (Supplementary Fig. S1). This revealed that the frequencies of most other immune cells in the patient were similar to those of healthy controls (Supplementary Fig. S2). In the patient, total CD4^+^ T cells and frequencies of the CD4^+^ subsets Th1, Th1*, Th2, Th17, T follicular helper, and regulatory T cells were not different from the healthy controls. Memory subsets of CD4^+^ T cells, including central memory, effector memory, and TEMRA, were also similar between the patient and healthy controls (Supplementary Fig. S2). Similarly, total CD8^+^ T cells and CD8^+^ T cell memory cells were not different between the patient and healthy controls (Supplementary Fig. S2). There were no differences between classical, nonclassical, and intermediate monocytes. pDCs and myeloid dendritic cells also had similar frequencies between the patient and healthy controls. Finally, total innate lymphoid cells showed no differences between the patient and healthy controls (Supplementary Fig. S2). While there are no differences in these immune cell populations, the patient has an unusual clinical presentation that cannot be fully explained by the reduced natural killer cells or reduced class-switched memory B cells seen from immunophenotyping (Figs 3 and 4). According to the Graham criteria, which includes histological features and the patient’s response to treatment, the patient presents with a possible *Mycobacterium tuberculosis* infection [15]. The study of the IEI known as Mendelian susceptibility to mycobacterial disease (MSMD) has revealed the crucial and non-redundant role of interferon-γ (IFN-γ) in antimycobacterial immunity [42]. In nearly all known MSMD-causing mutations, IFN-γ production or response is disrupted, suggesting that, despite normal frequencies of T and myeloid cell populations in our patient, ZBTB24 deficiency most likely causes a functional defect in IFN-γ-mediated immunity [42–47]. Therefore, there must be a functional defect in an immune population of the patient that is not reflected in its frequency and leads to susceptibility to *M. tuberculosis*. To investigate how ZBTB24 deficiency may lead to increased susceptibility to mycobacterial infections, we mined differentially methylated genes in ICF2 patients for genes known to cause MSMD or monogenic tuberculosis when mutated [47] using publicly available data from Velasco et al. (NCBI GEO database, accession GSE95040) [17, 48]. We found that several of these genes were hypomethylated in ICF2 (Supplementary Fig. S3). This could influence the expression of these genes and consequently result in increased susceptibility to mycobacterial disease. Therefore, while the patient does not have differences in the frequency of most immune populations except NK cells and class-switched memory B cells, our data suggests that ZBTB24 plays a role in the patient’s *M. tuberculosis* infection.

### Clinical phenotypes of ZBTB24 deficiency

To date, over 50 ICF2 patients with *ZBTB24* mutations have been reported (Table 1). Findings from these studies highlight the heterogeneous phenotypes of this disease. Clinical features that are common between cases include recurrent respiratory infections, hypertelorism, facial dysmorphism, and hepatomegaly (Table 1). The prominent immunological features of ICF2 patients are hypogammaglobulinemia of all or some antibody isotypes, reduced memory B cells, and reduced natural killer cells (Table 1). A fraction of patients also exhibited reduced T cell proliferation or a reversal of the ratio of CD4^+^ T cells to CD8^+^ T cells (Table 1). These findings are consistent with the clinical and immunological features of our patient, who exhibited reduced class-switched memory B cells and reduced NK cells. While these aspects of ICF2 are seen in many patients, most cases do not include every clinical or immunological feature listed. For example, susceptibility to Epstein–Barr virus (EBV), cytomegalovirus (CMV), and other viruses are reported in only a few cases, while most other cases report respiratory bacterial infections [7, 25, 32, 33, 35, 38] (Table 1). Another case also reports unusual mycobacteriosis, which is not found in any other patient [33]. Overall, while some immunological features of ZBTB24 deficiency are common between cases, the clinical phenotypes remain variable.

## Discussion

In this study, we characterized a novel homozygous pathogenic variant in *ZBTB24* and its functional consequences on the architecture of the immune system. We showed that the mutation Q375Hfs*3 results in a truncated protein due to a premature stop codon (Figs 1 and 2). Through bisulfite sequencing, we also demonstrated that this mutation disrupts the function of ZBTB24 in DNA methylation (Fig. 2). Identifying novel mutations is essential to the study and diagnosis of IEIs. IEIs are historically challenging to diagnose due to their widespread range of clinical manifestations. Additionally, IEIs are usually not the initial diagnosis a patient may receive when presenting with symptoms unrelated to immune dysfunction [49]. Consequently, patients may visit many specialists to address different symptoms, leading to delayed diagnosis or misdiagnosis [49]. It is therefore important to report novel mutations that result in IEIs to expand the known causes of these diseases. As more mutations are characterized, especially with functional studies, more diagnoses, and ultimately faster treatment, for patients with IEIs can be achieved. Characterizing the unique clinical manifestations of this mutation in *ZBTB24* will also help to identify other patients with ZBTB24 deficiency. The heterogeneous nature of this syndrome contributes to the difficulty of diagnosis, and it was recently reported that ICF syndrome cannot be identified through screening of T cell receptor excision circles (TRECs) at birth [50]. Therefore, reporting more ways ICF2 can manifest may aid in future diagnoses and faster treatment. This study also provides a comprehensive summary of ZBTB24 deficient patients reported in the literature, providing consolidated information about the consequences of ZBTB24 deficiency.

In-depth immunophenotyping of the patient using mass cytometry (cytometry by time-of-flight, CYTOF), computational methods, and manual gating revealed that ZBTB24 is critical for NK cell development or survival and the differentiation of class-switched memory B cells (Figs 3 and 4). This study was the first time in-depth immunophenotyping of an ICF2 patient was performed using CyTOF. The wide array of surface markers available for CyTOF combined with computational tools to cluster immune populations allowed us to examine the complex immune population structure of a patient with ZBTB24 deficiency. Not only did this confirm the immunological characteristics of previous ICF2 patients, but it also aided in the characterization of this novel *ZBTB24* mutation. Additionally, CyTOF allowed us to determine that other immune populations of the patient showed no difference in frequency when compared to healthy controls, meaning there may be some other functional defect resulting in abnormal immune manifestations. The limitation of using these immunological tools with only one ICF2 patient should not be ignored, and in-depth immunophenotyping of additional patients would provide a more complete understanding of how ZBTB24 deficiency shapes the immune system. Our characterization of all immune populations through CyTOF expands the current knowledge of the immune consequences of ZBTB24 deficiency, aiding in future studies and diagnosis of ICF2.

One of the most prominent features of ICF syndrome is decreased antibody production due to reduced class-switched memory B cells [8, 20, 22, 23, 27, 33–35, 38]. One possible explanation for this phenotype is the role of ZBTB24 in DNA repair. Helfricht et al. found that ZBTB24 deficiency in ICF2 syndrome leads to alternative NHEJ, suggesting that ZBTB24 regulates the repair of double-strand DNA breaks. NHEJ is integral to successful class switching in B cells, indicating that impaired class switching in ZBTB24 deficiency may result from defective NHEJ [12]. ZBTB24 has also been implicated in B cell proliferation. It has been shown that *ZBTB24* knockdown blocks the G0/1- to S-phase transition of the cell cycle in B cells, negatively impacting proliferation. *ZBTB24* knockdown also increased two negative regulators of B cell proliferation, IRF-4 and Blimp-1 [13]. These findings may offer a mechanism explaining the total B cell reduction in several ICF2 patients [7, 8, 18, 22, 24, 27–29, 32–35, 38, 39]. Together, these studies provide insight into reduced antibody levels in ZBTB24 deficiency due to reduced total and class-switched B cells, such as the reduction in class-switched memory B cells seen in our patient (Fig. 4).

Throughout the reported ICF2 patients, there is heterogeneity across immune phenotypes (Table 1). This may be due to the progressive nature of ICF syndrome, which has been reported in several patients [24, 33, 41]. The gradual depletion of B and T cells may partially explain phenotypic differences between ICF patients of different ages. Our patient, while having reduced frequencies of class-switched memory B cells, does not show complete impairment of antibody class switching (Fig. 4). This is consistent with other ICF2 patients, who demonstrate reduced levels of some antibody isotypes but not others [7, 8, 20–22, 24, 25, 27, 32, 33, 37, 38] (Table 1). Progressive depletion of immune populations may explain this discrepancy between patients, where patients with more advanced disease experience more severe immune phenotypes. However, several patients present with severe hypogammaglobulinemia early in life [5, 7, 8, 18, 19, 28].

Several immune phenotypes of ZBTB24 deficiency are not fully understood. Our patient and other ICF2 patients show reduced NK cells or NK cell activity due to ZBTB24 deficiency [22, 24, 27, 28, 33, 35, 38, 39] (Fig. 3; Table 1), yet the mechanism of how ZBTB24 contributes to NK cell biology is unknown. Along with heterogenous immune manifestations, ZBTB24 deficiency also leads to unusual clinical presentations in some patients. While bacterial respiratory infections are common in this syndrome (Table 1), some patients, including the subject of this study, have unique infections with intracellular pathogens. Our patient presents with a possible *M. tuberculosis* infection, while one other reported patient exhibited unusual mycobacteriosis [33]. Some patients also showed susceptibility to viral infections or high EBV or CMV viral loads [7, 25, 32, 33, 35, 38] (Table 1). While susceptibility to viral infections is a hallmark of NK cell deficiency (NKD), it can have a variable clinical presentation, with about 60% of patients with NKD presenting with herpesvirus infections [40]. This may explain why some, but not all, reported ICF2 patients with reduced NK cells suffer from viral infections. Regardless, these clinical phenotypes suggest a potential functional defect in immunity against intracellular pathogens. While NK cells are critical for immunity against such pathogens [51], the defect in NK cells seen in our patient and other ICF2 patients cannot fully explain these unusual infections. CD8^+^ T cells and CD4^+^ T cells are also critical to clearing intracellular pathogens. For intracellular bacteria such as *mycobacterium*, Th1 and Th1* cells are essential for activating phagocytes to effectively kill the pathogen [42–46]. Macrophages and monocytes themselves are also crucial to killing intracellular bacteria through IFN-γ signaling, as illustrated by impaired IFN-γ pathways in MSMD [42, 43, 46, 47]. Viruses replicating in the cytosol are often combatted by cytotoxic CD8^+^ T cells and NK cells [51, 52]. Therefore, ZBTB24 deficiency may contribute to a defect in immunity against intracellular pathogens that is not reflected in immune population frequency.

The patient’s presentation with granulomas raises the possibility of ICF2 being a granulomatous disease. Granulomas can be triggered by a variety of agents, and non-infectious granulomas have been observed in other IEIs, especially those causing defects in DNA damage repair [53]. However, the granulomas described in this patient were secondary to the possible *M. tuberculosis* infection reported. Therefore, ZBTB24 deficiency should not be considered a granulomatous disease on the basis of this patient. Additionally, IEI and ICF2 patients live in different parts of the world and are exposed to various environments. Therefore, the infections seen in our patient could be attributed to our patient coming from an *M. tuberculosis* endemic region. This may also help explain the heterogeneity in clinical presentation seen in ICF2 patients, where certain infections are more common in specific areas of the world. Regardless, our findings indicate that ICF2 should be included in IEIs associated with *M. tuberculosis*.

This study identified and characterized a novel mutation in *ZBTB24*. We expanded the current knowledge of how ZBTB24 deficiency impacts the structure of immune populations and corroborated the importance of ZBTB24 to immune function.

## Supplementary Material

uxaf016_suppl_Supplementary_Materials

## Data Availability

The data supporting the findings will be made available by the corresponding authors upon reasonable request.
