# Supplementary material for: In-depth immune profiling of a patient with immunodeficiency, centromeric instability, and facial anomalies syndrome type 2 caused by a novel mutation in ZBTB24
Source: Clin Exp Immunol. 2025 Mar 19;219(1):uxaf016. doi: 10.1093/cei/uxaf016 (PMC12062961; doi:10.1093/cei/uxaf016)
Supplement: uxaf016_suppl_Supplementary_Materials [file uxaf016_suppl_supplementary_materials.pdf]

Supplementary Material

Figure S1

Figure S2

Figure S3

Table S1

References

FIGURES

Figure S1. Manual gating strategy.

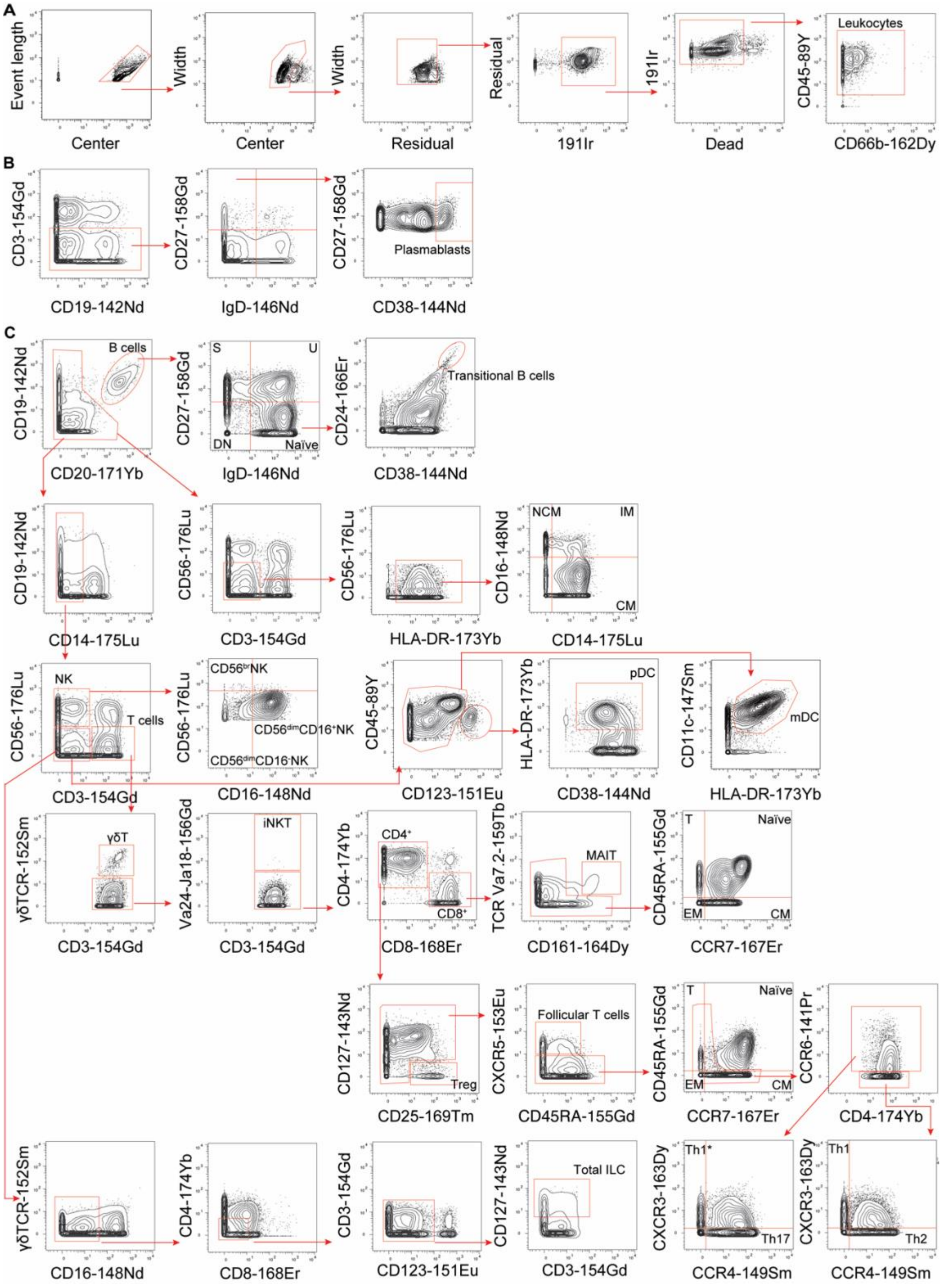

Figure S1. Manual gating strategy. (A) Pre-gating strategy performed before analysis in R. **(B)** Plasmablast gating strategy continuing from (A). **(C)** Manual gating strategy continuing from (A) used to identify other immune populations. S is switched, U is unswitched, DN is double negative, NCM is nonclassical monocytes, IM is intermediate monocytes, CM is classical monocytes, EM is effector memory, CM is central memory, and T is TEMRA.

Figure S2. Immunophenotyping frequencies by manual gating.

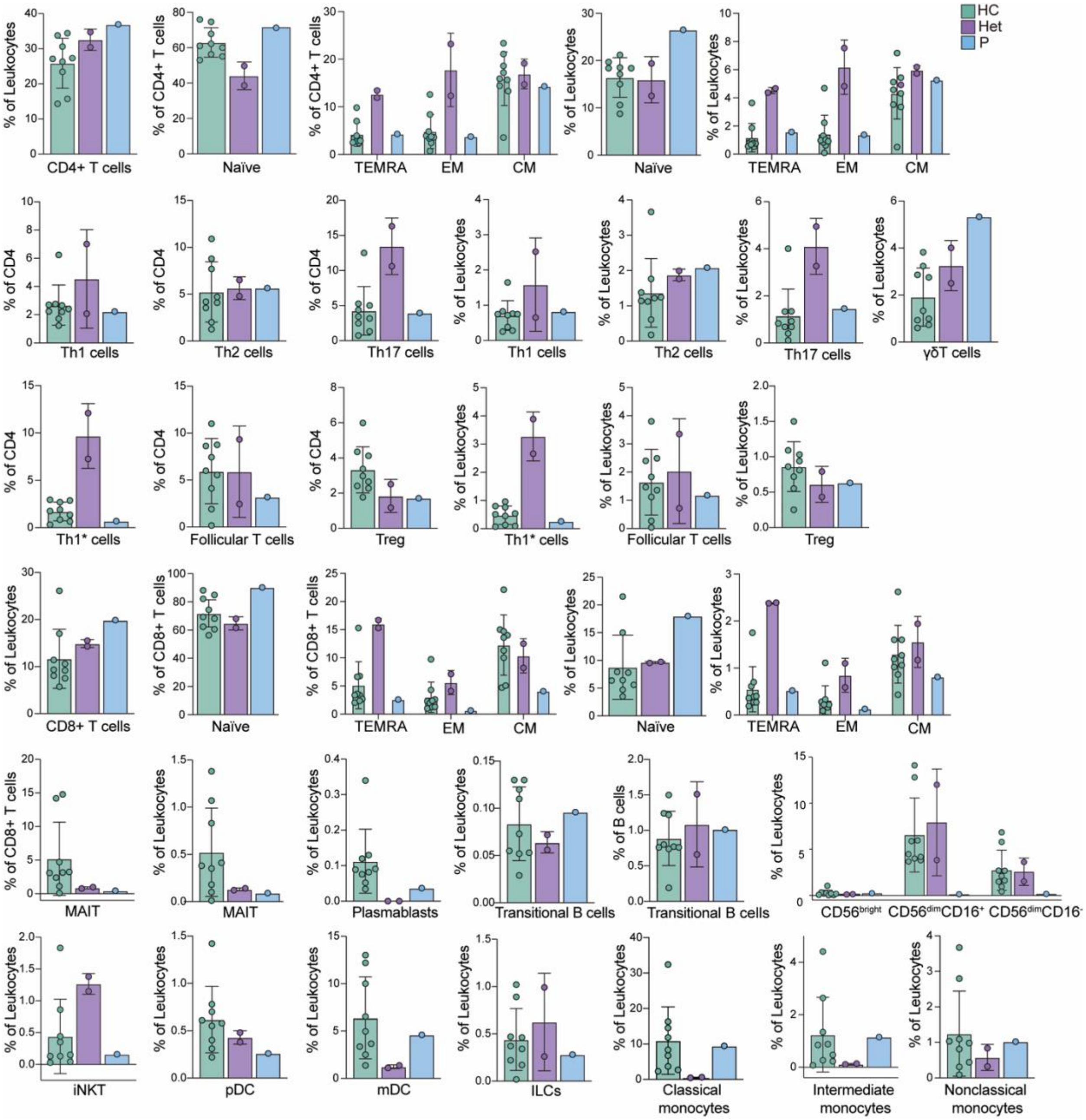

Figure S2. Immunophenotyping frequencies by manual gating. Frequencies of immune populations as a percentage of leukocytes or parent populations in healthy controls (HC), heterozygous carriers (Het), and the patient (P) following the manual gating strategy (Figure S1).

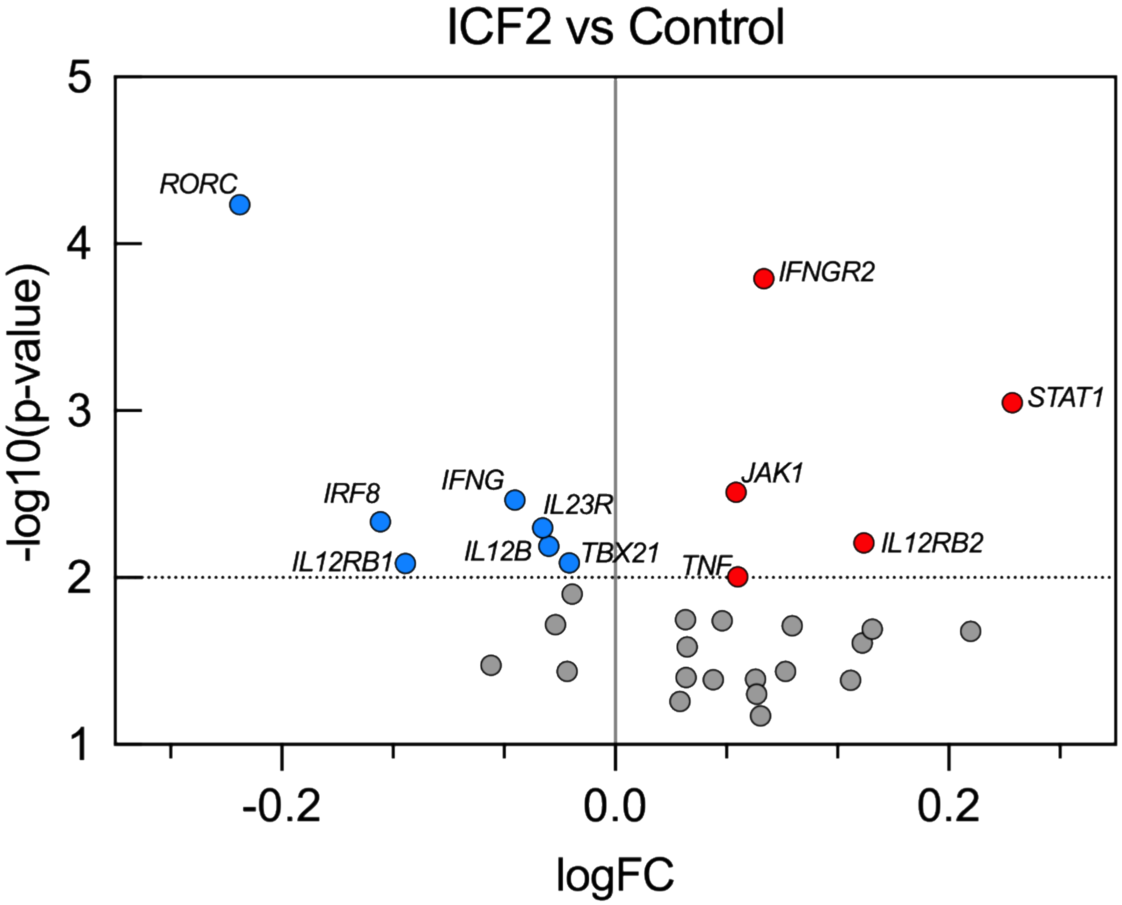

Figure S3. Highlighting genes that cause Mendelian Susceptibility to Mycobacterial Disease or Monogenic Tuberculosis when mutated in differential methylation data from ICF2 patients. Differential methylation of reported MSMD and monogenic TB genes was mined from comparative methylome data on ICF patients (NCBI GEO database, accession GSE95040)[1,2]. Genes that were differentially methylated in ICF2 patients compared to healthy controls were extracted from the data if they had an adjusted p-value of less than 0.05.

TABLES

Table S1. Primers

Target	Forward primer (5'-3')	Reverse primer (5'-3')	Method used
ZBTB24	CTCAGGACAGAAGTCTTT	AAAGACTTCTGTCCTGAG	Site-directed mutagenesis
ZBTB24	CACTCAGGACAGAAGTCTTTTA	TAAAAGACTTCTGTCCTGAGTG	Site-directed mutagenesis
ZBTB24	ATGGCAGAAACATCGCCAGAG	TCAGTGGTGATGGTGATGATGGCTCTGCTCCTGGCCA	His tag addition
ZBTB24	CAAGGCTCTGACCACCAAGC	GTGGCCTGTATGAACTCGGT	RT-qPCR
GUS	CTACTTGAAGATGGTGATCG	CTGTTCAAACAGATCACATC	RT-qPCR
SNORD115-14	GTTTGTTGTTGGGGGTTATTTT	CACATAATACCCTCTCCTTTCCTTA	Bisulfite sequencing
ZBTB24	CGCTTGTATCATTTGTAGAAAATG	ATACCCTATGTGAACGATATGT	Genomic DNA Sanger sequencing
T7 universal primer	TAATACGACTCACTATAGGG		Plasmid Sanger sequencing
ZBTB24 plasmids (pEZY3, pcDNA3.1)	TATCCTGCTGGAATTTATCTACA		Plasmid Sanger sequencing
ZBTB24 plasmids (pEZY3, pcDNA3.1)	GTTCAGAATAGACAAAACTTTGTG		Plasmid Sanger sequencing
ZBTB24 plasmids (pEZY3, pcDNA3.1)	GTCTTTAAGTACAATCACTTTTTAGC		Plasmid Sanger sequencing

ZBTB24 plasmids (pEZY3, pcDNA3.1)	CAGGTGAGAAGCCATTTAC	Plasmid Sanger sequencing
ZBTB24 plasmids (pEZY3, pcDNA3.1)	CTTCAGCTACAGCCATATCA	Plasmid Sanger sequencing
ZBTB24 plasmids (pEZY3, pcDNA3.1)	GAAACACTGGAACATCTTCAT	Plasmid Sanger sequencing

References

1. Velasco G, Grillo G, Touleimat N *et al.* Comparative methylome analysis of ICF patients identifies heterochromatin loci that require ZBTB24, CDCA7 and HELLS for their methylated state. *Hum Mol Genet* 2018;**27**:2409–24.

2. Edgar R. Gene Expression Omnibus: NCBI gene expression and hybridization array data repository. *Nucleic Acids Res* 2002;**30**:207–10.
